# Meningioma Related Epilepsy- Pathophysiology, Pre/postoperative Seizures Predicators and Treatment

**DOI:** 10.3389/fonc.2022.905976

**Published:** 2022-07-04

**Authors:** Rasha Elbadry Ahmed, Hailiang Tang, Anthony Asemota, Lei Huang, Warren Boling, Firas Bannout

**Affiliations:** ^1^ Department of Neurosurgery, Loma Linda University Medical Center, Loma Linda, CA, United States; ^2^ Department of Neurosurgery, Huasha Hospital, Fudan University, Shanghai, China; ^3^ Department of Physiology and Pharmacology, Loma Linda University, Loma Linda, CA, United States; ^4^ Department of Neurology, Loma Linda University Medical Center, Loma Linda, CA, United States

**Keywords:** meningiomas, seizure, epilepsy, risk factor, surgical resection, anti-seizure medications

## Abstract

Meningiomas are the most common primary brain tumors accounting for about 30% of all brain tumors. The vast majority of meningiomas are slow-growing and of benign histopathology rendering them curable by surgery alone. Symptomatic lesions depend on the location with signs of mass effect or neurological deficits. Seizures are the presenting symptoms in approximately 30% of cases, which negatively affect quality of life, limit independence, impair cognitive functioning, as well as increase the risk for psychiatric comorbidities including depression. Although surgical resection may offer seizure freedom in 60-90% of meningiomas, seizures persist after surgical resection in approximately 12-19% of patients. Anti-seizure medications (ASMs) are employed in management, however, are limited by adverse neurocognitive side-effects and inefficacy in some patients. The potential predictors of pre- and post-operative seizures in meningioma patients have been identified in the literature. Understanding various factors associated with seizure likelihood in meningioma patients can help guide more effective seizure control and allow for better determination of risk before and after surgery.

## Introduction

Meningioma accounts for about 30% of primary brain tumors and approximately 54% of primary benign ones (1–3). The vast majority of meningiomas are slow-growing and of benign histopathology (i.e., World Health organization (WHO) grade I tumors), rendering them curable by surgery alone (4, 5). Symptomatic lesions depend on the location with signs of mass effect or neurological deficits. Seizures are the presenting symptom in approximately 30% of cases, and in some studies, the percentage ranges from 13-60% (6–8). Although surgical resection can offer seizure freedom in 60-90% of meningiomas, seizures may persist after surgical resection in about 12-19% of patients (9, 10). Seizures can negatively affect the quality of life, hindering a patient’s independence, cognitive functions, and ability to drive safely (11–13). It puts patients at increased risk for different psychiatric comorbidities, including depression (14). Seizure control using various anti-seizure medications (ASMs) is usually offered despite adverse side effects on neurocognition and inefficacy in some patients (15).

Many theories have been postulated to explain the pathogenesis of brain tumor-related epilepsy (BTRE) in various brain tumors; however, unanswered questions remain regarding seizure control and management in meningioma patients, for example, the ability of surgical resections to cure seizures, when to start ASMs, duration of treatment as well as structured guidelines for patient selection for ASMs. Understanding and predicting seizures in meningioma can help guide seizure control and allow for better determination of at-risk patients before and after surgery. This review aims to summarize the pathogenesis of seizures in meningioma, pre- and post-operative predictors of seizures, surgical resection resulting in seizure freedom, the benefit of ASMs usage, intraoperative electrocorticography (ECoG) and electroencephalogram (EEG) monitoring in meningioma patients and proper patient selection.

## Incidence of Epilepsy in Meningioma

The incidence of pre-operative seizures in meningioma was respectively reported to be 29% of 4709 patients (7) and 14% of 598 patients (16) with supratentorial meningioma. Seizure freedom was achieved in about 69% of patients after surgery with 12% of new seizures onset postoperatively (17). Chozick’s study reported 63/158 patients with meningioma had pre-operative seizures and 40 (63.5%) of the 63 patients had complete resolution of seizure after surgery within follow-up years of 7.3± 3.8. In this cohort, 100% of 63 patients were on anti-seizure medications anticonvulsant preoperatively and during the initial stage postoperatively. The authors did not report the exact portion of these 43 patients weaned from anti-seizure medication over time postoperatively. While some neurosurgeons tended to stop using the medication approximately 6 months after surgery if there was no evidence of seizures, the other neurosurgeons continued using the anticonvulsant medications prophylactically. They reported that eighty-five patients (53.8% of 158) were eventually weaned from anticonvulsant and 44.7% were not off anti-seizure medications at the last follow-up visit postoperatively. Seizures recurred in 1 patient during weaning off ASMs process, in 4 patients with subtherapeutic ASMs levels, in 6 patients who were not on ASMs, in 2 patients correlated with alcohol abuse, and 5 patients with tumor recurrence. Eight patients (5.1%) with no history of preoperative epilepsy developed postoperative seizures. Chozick et al. concluded that in their study only the extent of tumor removal was a significant predictor of postoperative seizures. However, a history of preoperative seizures, preoperative language disturbance, postoperative anti-seizure medications status, postoperative hydrocephalus, or parietal region location of tumor were also predictive factors of the occurrence of postoperative seizures (18). Wirsching reported 26.6% of postoperative seizures within median 67 months (95% CI: 63–72) of post-surgery follow-up (19). The incidence of *de-novo* seizures in seizure naïve patients ranges widely from 2.4 to 19.4% (7, 17–23).The wide variation of these studies can be contributed to lacking standardization of retrospectively collected data from patients with different demographics, different features/locations/type of meningiomas, different follow up periods, different age groups analysis between pediatric and adult patients, and different surgical skills and techniques at different institutions. The majority of postoperative seizures were experienced in the first week after surgery, but one-third of patients experienced seizures three months after surgery (17).

## Pathogenesis of Epilepsy in Meningioma

The pathophysiology of brain tumor related epilepsy is multifactorial and can be divided into morphologic, biochemical and metabolic causes. The morphologic changes in the peritumoral neocortex include the connection of the neurons and the connectivity and localization of the synaptic vesicles, causing higher concentration of voltage-dependent Na+ channel, Ca++ and Glutamate receptors with loss of inhibitory synapses and an increase of the excitatory synapses. Biochemically, there is an increase of Glutamatergic and reduction in GABAergic somatostatin immunoreactive neurons. At the ion level, there is a report of low Mg2+, high extracellular K+, high Fe3+, low neuron-specific K+/Cl− cotransporter-2 (KCC2). Extracellular peritumoral pH is thought to be slightly alkaline. Finally there are enzymatic, amino acid and immunologic changes with upregulation of Glutamatergic receptors for NMDA and AMPA neurotransmitters (24). More recently, the genetic drivers of epileptogenicity in meningiomas have been investigated. NF2 mutation was shown to be predictive marker for preoperative seizures, which was *via* an indirect mediation effect with atypical histology and edema (25). Meningioma originates from arachnoid cap cells and is usually a slow-growing tumor (1). Such slow growth can partially explain the peritumoral changes that lead to epileptogenicity (24, 26). The partial differentiation of cortical brain surface may produce an epileptogenic zone, thus causing denervation hypersensitivity (27). The morphologic changes that develop in the brain tissue adjacent to the lesion, like inefficient neuronal migration, synaptic vesicles, and glial gap-junction coupling alterations, are also thought to contribute to seizure generation (28). Although pediatric meningioma is rare, epilepsy was reported as one of common symptoms (29). Inefficient neuronal migration may serve as an additional peritumoral mechanism of epileptogenesis in this age group of patients.

The percentage of brain edema in patients with meningioma ranges between 30% to 60% (30–32). It is usually vasogenic and related to an increase in pial supply, angiogenesis, and increased expression of vascular endothelial growth factor (VEGF) (33, 34). Chemical changes in the peritumoral milieu and local hypoxia from local tumor compression are thought to be underlying mechanisms that decrease the threshold for seizures (26). Increased levels of glutamate in the peritumoral edema are often described as an instigating factor for the state of hyperexcitability and epilepsy (8, 26). Edema is strongly correlated with brain invasion (35), and may also be intimately associated with tumor location and more invasive and higher grades of meningioma (31, 32, 36). Notably Hess et al. reported a five-fold increase in edema volume in patients with brain invasion compared to those without, with a reported 20% increase of risk of brain invasion with each 1cm increase in peritumoral edema (35). Chernov, et al. reported a high incidence of peritumoral edema in macroscopically invasive meningiomas (37). Brain invasion and breakdown of the arachnoid layer distort and alter the peritumoral cortex, releasing amino acids and affecting the neurotransmitter pathway (35, 38).

For post-operative seizures onset, intraoperative strong adhesions, the need for microdissection, and possible injury to cortical surface and irritation can contribute to the generation, especially in seizure naïve patients (36). Retraction and manipulation, which are sometimes necessary to achieve total resection in skull base lesions, can also lead to further cortical damage and edema (39). Post-operative complications like infection, hematoma, and hydrocephalus can further increase cerebral edema and increase the risk of seizures (40).

Based on histopathological characteristics, the WHO grading system classifies meningiomas into grade I (benign), grade II (atypical), and grade III (anaplastic) (41). Hess et al. analyzed the brain invasion and risk of seizure retrospectively in a total of 176 patients with meningioma. There were 92 (52%) grade I, 79 (45%) grade II, and 5 (3%) grade III tumors. Grade I meningioma included 16 (17%) transitional, 4 (4%) secretory, 68 (74%) meningothelial, 3 (3%) fibrous, and 1 (1%) angiomatous subtypes. Preoperative seizures were present in 10 (11%) of 92 patients with grade I meningioma, 23 (29%) of 79 patients with grade II meningioma, and absence in patients with anaplastic meningioma. In grade I meningioma, histopathological subtype correlated significantly with the rate of preoperative epilepsy. Overall, the risk of preoperative seizures was significantly higher in patients with a grade II or III tumor than in those with a grade I tumor. Brain invasion was absent in all patients with a grade I meningioma, but it was present in 35 (44%) of those with an atypical and 3 (60%) with an anaplastic meningioma. Brain invasion was independent of tumor volume but strongly correlated with edema volume. Multivariate analyses showed the risk of preoperative seizures was increased distinctly in patients with brain-invasive meningioma over those with noninvasive meningioma (OR 5.26, 95% CI 1.52–18.15; p = 0.009). However, postoperative seizure-free rates were similar among patients with invasive and those with noninvasive meningioma. The incidence of postoperative epilepsy was correlated significantly with the increasing preoperative tumor volume (35). In another retrospective study, Gadot et al. reviewed the 384 patients who underwent meningioma resection. The significant association was not found between any histological subtype and worse postoperative seizure outcomes. However, there was an associative tendency between subtypes of higher grades (malignant, rhabdoid) with worse postoperative seizure outcomes. The subtypes of lower grades (fibrous, transitional) trended toward improved postoperative outcomes (p = 0.081) (25). There are no data in the medical literature for the incidental small meningioma, which are not part of the epileptogenic network.

## Predictors of Epilepsy in Meningioma

In an attempt to better understand and predict seizures in patients with meningioma, several retrospective studies investigated the possible predictors of seizures both pre-operatively and post-operatively. Throughout the literature, peritumoral edema and location have been associated with seizures in meningioma. Peritumoral edema has been extensively studied and considered the strongest predictor of seizure in both pre- and post-operative periods (7, 8, 17, 20, 21, 26, 35). There is a less likelihood of achieving seizure freedom postoperatively in patients with with significant pre-operative edema (21, 42).

### Preoperative Predictors

The preoperative predictors of epilepsy/seizures are summarized in **Table 1**.

**Table 1:** Predictors of preoperative epilepsy/seizures.

Reference	Study type	Summray of findings (bold texts indicating the proposed predictors)
Englot et al., 2016 (6)	A meta-analysis of 39 observational series cases (4709 patients surgically treated menigiomas) published between January 1980 and September 2014	The significantly predictors were **male sex, peritumoral edema and non-skull base location**
Seyedi et al, 2018 (17)	Retrospective cohort study of 295 patients that underwent resection of a supratentorial meningioma between 2007-2015.	Seventy-two (24.4%) of the patients experienced seizures preoperatively. **Peritumoral edema was a significant predictor of preoperative seizure**; headaches and neurological deficits were associated with decreased incidence of preoperative seizures.
Xue et al., 2018 (20)	A retrospective study of 113 consecutive adult (> 18 years old) patients with newly diagnosed meningioma underwent operation between 2006 and 2008 were included and followed up until the end of 2015.	A total of 21/113 (18.6%) patients experienced seizures before surgery. **Tumor diameter >/= 3.5 cm as a risk factor for preoperative seizures**, but presence of headache and skull base tumor location decreased the risk of preoperative seizures
Lieu and Howng, 1999 (22)	A retrospective study of a consecutive series of 222 surgically treated patients with meningiomas,	There were 59 (26.6%) of the patients presented epilepsy as their initial symptom. **Intracranial supratentorial or convexity meningiomas with evidence of severe peritumoral edema** significantly contribute to preoperative epilepsy.
Chen et al., 2017 (23)	Retrospective chart review of 1033 subjects undergoing resection of supratentorial meningioma bewtween1991 and 2014	Preoperative seizures occurred in 234 (22.7%) patients. The predictors of preoperative seizures: **presence of ≥1 cm peritumoral edema, non-skull base tumor location, older patient age.** Presenting symptoms of headache or cranial nerve deficit was associated with decreased odds of preoperative seizures. Non-skull base supratentorial meningiomas with surrounding edema have the highest risk for preoperative seizure.
Kawaguchi et al, 1996 (32)	A retrospective analysis of clinical symptoms and computed tomographic findings in 83 consecutive patients	Twenty sever (33%) patients presenting with epilepsy as the first symptom. **Peritumoral edema** is a significant epileptogenic factor associated with both cerebral convexity and parasagittal meningiomas.
Hess et al., 2018 (35)	Retrospective review of all patients with a histopathologically diagnosed primary meningioma underwent resection between 1991 and 2015.	In grade I meningioma, histopathological subtype correlated significantly with the rate of preoperative epilepsy. Overall, the risk of preoperative seizures in **meningioma grade II or III tumor was** significantly higher than in those with a grade I tumor. Brain invasion was absent in all patients with a grade I meningioma, but it was present in 35 (44%) of those with an atypical and 3 (60%) with an anaplastic meningioma. **Brain invasion is a strong predictor for preoperative,** but not postoperative, seizures. Although associated with increased peritumoral edema, seizures in patients with invasive meningioma might be related to cortical invasion.
Li et al., 2020 (43)	A retrospective study in 778 patients undergoing supratentorial meningiomas surgery between 2011 and 2012	A total of 100 (12.9%) patients experienced preoperative seizures. **Motor cortex involvement and peritumoral edema ≥ 1 cm** were significant risk factors of preoperative seizures. Presenting symptoms of headache, and age ≥ 55 years were associated with decreased incidence of preoperative seizures.
Hamasaki et al., 2012 (44)	A retrospective study restricted to patients with WHO grade I intracranial meningioma in database between 1968 and 2011, of which 44 patients with epilepsy were enrolled in (epilepsy group). The patients with WHO grade I meningioma without epilepsy were recruited consecutively from the database between 2007 and 2011, which resulted in 56 patients in the control group.	Preoperative recurrent epileptic seizures in 12.7% (88) patients. Voxel-wise comparison between 3D MRI scans obtained from patients with meningioma-associated epilepsy and those from control patients using spatial normalization techniques on neuroimaging data The highest incidence of epilepsy was seen with **tumors located on premotor cortex in the frontal lobe. Tumor diameter/volume and patient’s age were positive and negative predictors, respectively, for onset of epilepsy.**

The bold texts in Table 1 indicate the the predictors proposed in each study, respectively.

In a retrospective study by Li et al., peritumoral edema of > 1cm was among the risk factors identified for preoperative seizures in meningioma patients (43). Tumor location in the temporal, parietal, and frontal (adjacent to neocortex) lobes are more likely to be associated with seizures (7, 18, 20, 21). Specifically, Lieu and Howng noted that tumor located in the temporal lobe increased the risk of pre-operative seizures than other lobes. The increased peritumoral edema noticed in convexity and parasagittal meningioma is thought to favor the likelihood of increased seizure frequency in affected individuals. Non-skull base meningiomas are suggested to be more aggressive with a high MIB-index (percentage of immunoreactive tumor cells) which favors brain invasion, edema, and seizure (20, 45). In another study, no consensus was found regarding the most epileptogenic cortical area (46).

Most studies suggest that bigger tumors are naturally associated with a higher risk of seizure preoperatively. Conceivably, larger tumors can cause more irritation and compression on surrounding brain tissue. Similar results reported by Chen et al. showed that tumors larger than 3 cm in size, of higher grade with peritumoral edema more than 1 cm are associated with preoperative seizures (20). In one study, no statistically significant correlation between tumor size and preoperative seizures could be found (43), while mean tumor diameter of 3.5 cm was used at cut-off to demonstrate an association with postoperative in-hospital seizures.

Interestingly meningiomas are more common in females, but males are more likely to present with seizures. Many studies have shown the male gender as a risk factor for developing preoperative seizures (7, 8, 20, 23, 43). There is a possible association of male gender with higher grade meningioma, larger size, and more edema (20). Younger age was a predictor (44), and a lower incidence of preoperative seizures was found in meningioma patients older than 55 years old (43).

Other factor like preoperative Karnofsky score (KPS) were also studied. A KPS <80 was positively associated with pre-operative seizures (40). Englot et al. reported a decreased incidence of preoperative seizures in patients presenting with cranial nerve deficits (7). However, there are limitations in symptom frequency studies. Prospective studies are needed to validate these potential predictors.

### Postoperative Predictors

The postoperative predictors of epilepsy/seizures are summarized in **Table 2**.

**Table 2:** Predictors of postoperative epilepsy/seizures.

Reference	Study type	Summary of findings (bold texts indicating the proposed predictors)
Englot et al., 2016 (6)	A meta-analysis of 39 observational series cases (4709 patients surgically treated menigiomas) published between January 1980 and September 2014	Seizure freedom was achieved in 69.3% of 703 patients with preoperative epilepsy after surgery. Among patients with **preoperative seizures**, a strong association was observed between persistent postoperative seizures and **peritumoral edema. Tumor progression** after surgery was associated with seizure recurrence. Postoperative *de-novo* seizures were developed in 12.3% of 1085 patients. No difference in the rate of new postoperative seizures was observed with or without perioperative prophylactic anticonvulsants. Postoperative *de-novo* seizures were more common in those with **a history of previous radiation** or with **gross-total resection**. However, the total number of patients with new seizures in each of these categories was low (9–11 patients), limiting the ability to draw conclusions.
Seyedi, et al., 2018 (17)	Retrospective cohort study of 295 patients underwent resection of a supratentorial meningioma between 2007-2015.	Seventy-two (24.4%) of the patients experienced seizures preoperatively, and a complete seizure freedom was achieved in 63.9% of them. A total of 20.3% of the patients experienced seizures after surgery. Two hundred twenty three (75.6%) of the patients did not experience seizures preoperatively, but 15.2% of them developed postoperative *de-novo* seizures. Time to first seizure in patients who developed *de-novo* postoperative seizures was one week (47%), within one month postoperative (21%) and three months after surgery (32%). ASMs had a treatment success rate of 98.2% in preoperative seizures, and 98.0% in postoperative seizures. Postoperative seizures were **increased in left-sided meningiomas, and decreased with convexity/parasagittal/falx meningiomas as well as with absence of postoperative complications**.
Chozick et al, 1996 (18)	A retrospective access the incidence of postoperative seizures in 158 patients with supratentorial meningiomas diagnosed by computerized tomography (CT) and/or magnetic resonance (MR) imaging	Of 63 patients with preoperative seizures, 40 (63.5%) had complete cessation of seizures after surgery. Overall 88.9% of patients with preoperative seizures achieved complete seizure control postoperatively. The mean follow-up period was 6.4 ± 3.7 years with a minimum follow-up period of 2 years. Eight patients (5.1%) developed postoperative *de-novo* seizures during mean follow-up period of 5.7± 2.8 years. The onset of seizures occurred in conjunction with weaning from anticonvulsant medication in one patient, with subtherapeutic anticonvulsant medication levels in two patients, and with tumor recurrence in three patients; two patients experienced seizures while not receiving anticonvulsant medication. Predictors of postoperative seizures included: **preoperative seizure history, preoperative language disturbance, extent of tumor removal, parietal location of tumor, postoperative anticonvulsant medication status, and postoperative hydrocephalus**. Earlier detection and treatment of supratentorial meningiomas might improve seizure outcome in patients with preoperative epilepsy.
Wirsching et al., 2016 (19)	A retrospective study of 779 patients treated for histologically confirmed intracranial meningioma 2000 and 2013	Epileptic seizures occurred in 244 (31.3%) patients before surgery, of whom 144 (59.0%) became seizure-free after surgery. The follow up period was not reported. Of the 535 patients without preoperative seizures, 104 (19.4%) developed postoperative *de-novo* epilepsy. Predictors of postoperative epilepsy were **preoperative epilepsy, major surgical complications including CNS infections, hydrocephalus, re-craniotomy, and symptomatic intracranial hemorrhage, as well as postoperative epileptiform EEG potentials, younger age, and tumor progression.** Postoperative improvement or recovery from preoperative neurologic deficits is associated with improved seizure control.
Xue et al, 2018 (20)	A retrospective study of 113 consecutive adult (> 18 years old) patients with newly diagnosed meningioma underwent operation between 2006 and 2008.	A total of 21/113 (18.6%) patients experienced preoperative seizure, of whom 8/21 (38.1%) become seizure-free after surgery. The followed up period last until the end of 2015. Thirteen (14%) patients developed postoperative *de-novo* seizures. **Larger tumor size (diameter (>/= 3.5 cm) and preoperative seizures are associated with postoperative seizures.**
Morsy et al, 2019 (21)	A prospective study of 40 patients with intracranial meningioma Group A with preoperative seizures Group B with no preoperative epilepsy.	In Group “A”, 8 (40%) patients had good postoperative seizure control, 12 (60%) had poor seizure control. In Group “B” 3 (15%), patients developed postoperative *de-novo* seizures. **Postoperative complication was significantly associated with *de-novo* epilepsy** and poor seizure control.
Lieu and Howng, 1999 (22)	A retrospective study of a consecutive series of 222 surgically treated meningiomas,	A total of 52 patients had postoperative epilepsy. The follow-up periods ranged from 1 to 12 years. Among them, 22 patients (37.3%) had preoperative epilepsy which continued postoperatively. Surgical excision of the intracranial meningiomas stopped the epilepsy in about 62.7% of the patients. A total of 30 (13.5%) patients developed postoperative *de-novo* epilepsy, of which 18 are early onset of postoperative epilepsy (within 1 week) and 12 are late postoperative epilepsy (beyond 1 week). During the follow-up periods, 37 (71.2%) patients were seizure-free after 1 year of anticonvulsant therapy. Patients with **preoperative epilepsy, and tumors with evidence of severe perifocal edema or cerebral edema at the operative site** were significantly more likely to develop postoperative epilepsy.
Chen et al., 2017 (23)	Retrospective chart review of 1033 subjects undergoing resection of supratentorial meningioma between 1991 and 2014. Follow-up occurred through mid-2015.	Preoperative seizures occurred in 234 (22.7%) subjects. Fifty four (5.9%) patients experienced acute postoperative seizures prior to discharge (median duration of postop stay: 4 days, 5.72 ± 6.63), which significantly associated with **weakness as a presenting symptom, nonskull base location, and occurrence of medical/surgical complications.** During at least 1 year of postoperative follow-up, there were 51 (13.7%) of 373 patients had postoperative seizure after discharge. Of whom, 25 (2.4%) patients were *de-novo* postoperative seizures and 26 patients were with preoperative seizures. **The presence of preoperative, the occurrence of postoperative in-hospital seizures and medical/surgical complications were significant predictors of postoperative seizures after discharge.**
Gadot, 2021 (25)	A retrospective review of 384 patients underwent meningioma resection from 2008 to 2020.	Fifty-nine patients (15.4%) had preoperative seizures, of whom 57 had sufficient postoperative data to determine Engel class outcome. The median follow-up duration for patients with Engel class I outcomes was 14 months (range 3–26 months). Forty-two patients (74%) patients achieved Engel class I seizure freedom, with most achieving complete seizure freedom (Engel class Ia) at longest follow-up. The median follow-up duration was 20 months (range 6–34 months) for Engel class II–IV outcomes Eight (14%) patients experienced poor seizure control (Engel class IV), with the majority of those experiencing worsened seizure burden compared with preoperative baseline (Engel class IVc). ASM status at last follow-up was determined and revealed that 33 (59%) of patients were still taking at least 1 ASM at lengthiest follow-up, whereas 23 (41%) patients were not taking any ASMs. **Postresection ischemia, higher WHO grade, elevated MIB-1 index, and disease recurrence independently predict postoperative seizure.**
Lu et al., 2019 (40)	A meta-analysis, searches of 4 electronic databases from inception to February 2019, resulting 430 reports with 5681 patients with meningioma.	Independent predictors of postoperative seizures identified were: **preoperative seizure history, non-skull base location, postoperative complications, meningioma recurrence**.
Li et al., 2020 (43)	A retrospective study of 778 patients underwent supratentorial meningiomas surgery between 2011 and 2012.	A total of 100 (12.9%) patients experienced preoperative seizures, 41 patients (5.3%) experienced acute postoperative in-hospital seizures, and 91 (13.5%, n = 673) patients experienced postoperative seizures after discharge. The occurrence of any **medical/surgical complication** were significant risk factors for postoperative in-hospital seizures. Postoperative seizures after discharge were associated with **tumor maximal diameter >/= 3.5 cm, preoperative, postoperative in-hospital seizures and tumor recurrence/progression**. **Tumor recurrence/progression was the only predictor of *de-novo* postoperative seizures.** The probability of seizure freedom in the 5-year follow-up was roughly 59% among patients with preoperative seizures, and 87% among patients without preoperative seizures. The use of postoperative prophylactic ASMs did not reduce the incidence of seizures.
Zheng et al., 2013 (47)	A retrospective study of 97 patients with supratentorial meningioma plus preoperative seizures	Sixty-two of 97 patients (63.9%) were seizure free for the entire postoperative follow-up period (29.5 +/- 11.8 months), while 13 patients (13.4%) still had frequent seizures at the end of follow-up. Fourteen of 97 patients (14.4%) experienced early postoperative seizures, and emergence of new postoperative neurological deficits was the only significant risk. Thirty-three patients (34.0%) experienced late postoperative seizures at some time during follow-up, including 12 of 14 patients with early postoperative seizures. **Factors associated with late postoperative seizures included tumor progression and new postoperative neurological deficits.** Decreased cerebral/vascular injury intraoperatively may lead to fewer postoperative neurological deficits and better seizure outcome.

The bold texts in Table 2 indicate the the predictors proposed in each study, respectively.

The International League Against Epilepsy (ILAE) defined acute postoperative seizures as seizures happening within seven days of craniotomy (48). The late postoperative seizure is defined as on set of epilepsy beyond the first week of surgery (21, 49). In a retrospective study of 556 patients who underwent meningioma surgery, there were 74 patients with postoperative seizures, in which 43% was late seizures (49). Some studies categorized postoperative seizures into early, late, in-hospital, and post- hospital discharge. Identifying possible predictors of seizures postoperatively can help guide seizure control and minimize complications associated with ASMs long-term usage (13, 17, 20, 43, 47, 50).

Tumor location, size, grade, involvement of motor area and KPS have all been studied as predictors for postoperative seizures (21, 23). In one study, the occurrence of early in-hospital seizures was associated with involvement of motor cortex, post-operative KPS < 70, postoperative complications, and preoperative seizures (43). It was suggested that decreased threshold and the increased cortex sensitivity during the immediate postoperative period are important factors to be considered, and ASMs use may be justifiable in this period. The KPS < 80 was an independent predictor for postoperative seizures, with an almost threefold higher risk of having preoperative seizures (40). This further explains the impact of seizures on quality of life. Skull base lesions were associated with decreased incidence of seizures preoperatively, with an opposite trend and increased incidence in the postoperative period (40). Chen et al., in one study of 1033 patients, reported decreased incidence of seizure in non-skull base lesions (20). Skull base lesions require more brain retraction, further increasing brain edema (7, 51). Scott et al. noted an association of left-sided meningioma with greater risk for developing seizures (52), with higher rates of postoperative seizures reported on the left hemisphere (66.7%) compared to the right (23.3%) (17). In a radiological study analyzing 3D structural magnetic resonance imaging (MRI) of meningioma patients to identify hotspots for seizures, results showed a high likelihood of seizures when the lesion was located on the motor cortex of the frontal lobe (44).

Preoperative seizures were strong predictors of postoperative seizures, especially uncontrolled ones (13, 17, 20, 43). There is a contradiction in the literature regarding neurological deficits as presenting symptoms. In some studies, it was associated with less incidence of preoperative seizures (17, 20), and in others, it was found to be significantly associated with postoperative seizures before discharge (10, 19). On univariate analysis, Chen et al. found that a neurological deficit in the form of new weakness, pneumonia, hematoma, and infarction with edema were significantly associated with in-hospital seizures. In their study, weakness was a predictor for in-hospital but not pre-operative or post-discharge seizures (20). Interestingly, Wirsching et al. found that postoperative improvement and recovery from preoperative neurological deficits were associated with a lower risk of postoperative seizure and improved control (19).

Postoperative complications are independent predictors of postoperative seizures (20). In the immediate postoperative period, the brain is more sensitive with a decreased threshold for seizure (43). Any irritation to the highly sensitive and probably still edematous neocortex can aggravate seizures immediately after surgery. A positive correlation has been established between postoperative complications like hematoma, hydrocephalus, infection, and edema (40). Permanent new postoperative neurological deficits, especially in patients with vascular injury, increased the risk of seizures postoperatively significantly (47). Wirshing et al. specified major surgical complications like central nervous system infections, hydrocephalus, re-craniotomy, and symptomatic intracranial hemorrhage as risk factors for postoperative seizures (19).

For seizures after discharge, Li et al. identified tumor size > 3.5 cm, preoperative seizures, and tumor progression as strong predictors (43). In the same study, postoperative complications were associated with acute postoperative seizures, but no correlation with postoperative seizures on long-term follow-up. In another study, surgical complications were associated with in-hospital seizures and post-discharge seizures in seizure naïve patients (19, 53). Chen et al. did not find tumor recurrence or subtotal resection to be strong predictors for postoperative seizures (20). Englot et al. found a strong association of cranial nerve deficits with post-discharge seizure on univariate analysis (7).

## Surgical Resection and Epilepsy Freedom

Improved surgical techniques and earlier diagnosis of meningioma have affected the extent of resection with favorable outcomes. As previously reported, surgery offers seizure freedom in 70% of patients with rates ranging between 19% to 90% (7, 21). In some studies, the overall seizure freedom over a 5-year follow-up was 87% in patients with preoperative seizures and 59% in seizure naïve patients (4, 43). Lu et al. reported a 30-40% postoperative seizures in patients with seizure history preoperatively and 10-15% in seizure naïve patients (40). Komotar et al. showed a significant influence of gross total resection on seizure rates (54). These reports support surgical intervention and cytoreduction in patients with persistent seizures. In contrast, new postoperative seizures were reported to occur more frequently in patients with gross total resection (46). A possible explanation is that greater manipulation, dissection, and retraction of the brain to achieve gross total resection, can cause cortical injury, irritation, edema, and seizures. In one study, Simpson grade I resection was correlated with postoperative seizures (39). Most of these lesions were convexity meningiomas, which strongly correlate with seizures. Therefore, Simpson grading was not clinically relevant in that study. Similar results were reported by Hess et al., with no statistical significance noted between Simpson grade and postoperative seizures (35). Multiple studies showed an association between seizure and tumor recurrence/progression (23, 47). One postulated theory is that there is possible reactivation of previous epileptogenic focus or formation of a new one with tumor recurrence (40, 43). WHO grade I lesions have low recurrence rates, and with gross total resection, this can be a protective factor against postoperative seizures (4, 5).

Most of the data in the literature report seizure freedom after craniotomy and resection, with few studies discussing other treatment modalities like radiosurgery. Kondziolka et al. reported one case of mortality without further details (54). In Zada’s study of 116 patients undergoing Gamma knife for meningioma, there were zero seizure rates over 75 months of follow-up (55). Pollack et al. reported a 1.6% rate of new or worsened seizures after radiosurgery (56). Decreased seizure freedom rates have been reported after surgery in patients with intractable seizures preoperatively (40).

## Management of Uncontrolled Meningioma Related Epilepsy; Medications and Epilepsy Surgery

The American Academy of Neurology does not recommend the prophylactic use of ASMs in newly diagnosed brain tumors. In our institution we do not advocate for obtaining EEG pre-operatively to help in determining placing patient on ASMs. Yet some surgeons advocate for prophylactic use of ASMs in the immediate postoperative period to prevent *de-novo* seizures (57). In one study by Zheng et al., ASMs reduced the risk of early postoperative seizures (8, 58). ASMs can be used in patients with preoperative seizures as a temporizing measure until surgical resection. It is estimated that 40% of patients with well-controlled seizures before surgery could be weaned off ASMs over 27 months postoperatively, and only 22% remained with intractable seizures (8). For better patient selection and ASM use postoperatively, the STAMPE scoring system was an attempt to help guide epilepsy treatment in meningioma patients (19). They suggested a simple scoring system comprised of possible risk factors like sensorimotor deficit, tumor progression, age < 55 years, major surgical complication, preoperative seizures, post-operative EEG, and brain edema. Results were however not statistically significant and needed further validation.

Evaluation for epilepsy surgery for further resection after delineating the epileptogenic zone by intracranial EEG monitoring (grids, strips, or stereotactic electrodes) including intraoperative ECoG has been the gold standard approach in Level 4 epilepsy centers for patients with lesional epilepsies who have failed at least two adequately selected and dosed ASMs. EEG can be helpful for the assessment of seizure recurrence upon weaning or withdrawal of ASMs. Multiple studies suggested routine uses of EEG postoperatively to predict seizure recurrence. In one study of 340 patients, epileptiform discharge predicted postoperative seizures, advocating for routine EEG postoperative use (19). Intraoperative ECoG mapping and resection of secondary seizure focus in the peritumoral cortex can increase rates of seizure freedom postoperatively (23, 27). Postoperative EEG with epileptiform discharges is suggested as predictor for postoperative seizure occurrences (19, 59). However, the American academy of Neurology published a guideline practice in adult patients with epilepsy who achieved seizure freedom (though not specifically for meningioma), ordering EEG to detect interictal epileptiform discharges is not helpful to guide the decision of ASMs continuation. However, higher confidence of this approach exists in pediatric patients. An epileptiform potentials on EEG in pediatric patients increases the risk of seizure recurrence (60).

In our center we assess every patient with meningioma related epilepsy, particularly patients who continue to have uncontrolled postoperative seizures with stereotactic depth electrodes (S-EEG) implantation or by subdural grid/strip electrodes, and in cases where functional mapping is essential to rule out the involvement of eloquent cortex of the epileptogenic zone. S-EEG provides a safer option for patients who are planned to have a second surgery knowing the expected challenges from prior surgery complications like adhesions, infections, bleeding etc. Gross functional mapping can also be performed by S-EEG comparing to detailed functional mapping by grid/strip electrodes. In areas where there is room for safer resection outside eloquent cortices, S-EEG is helpful to encompass the surrounding edges of the lesion and for reaching remote areas of interest as well, like mesial temporal structures to rule out dual pathology.

**Figure 1:**
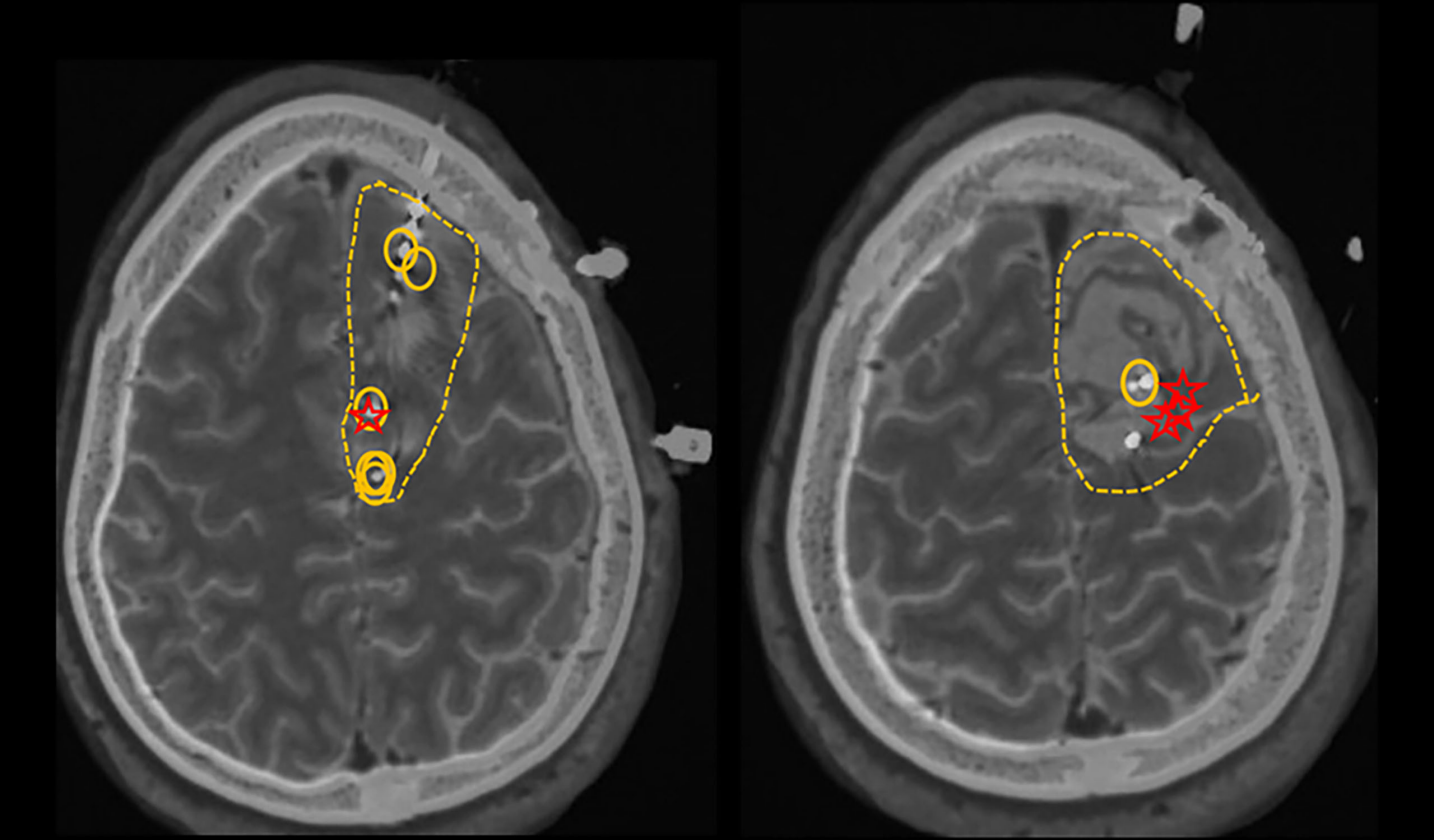
Circles represent active interictal epileptiform discharges (the irritative zones). Stars represent the first involved contacts at the ictal onset. Dashed lines represent the proposed resection zone.

The following case illustrates our own experience in post-operative seizure management after meningioma resection. A 36-year-old left-handed male who underwent left midfrontal parasagittal superior larger meningioma (6 x 7 cm) resection developed new-onset seizures 8-10 months postoperatively. His 3-month post-surgical MRI showed complete resection of the tumor. Approximately 11 months after the resection he developed his first ‘tonic-clonic’ seizure. It started with right-sided numbness, weakness and tingling of his back going down his mid-spine. He was subsequently started on Lamotrigine, but continued to experience repeated seizures which started with the same tingling sensations down to his spine, coupled with abnormal butterfly sensations in his abdomen, ultimately culminating in right foot shaking movements, with further spread to his right arm. Due to developing drug resistant epilepsy (DRE) including lamotrigine, lacosamide and levetiracetam, he underwent further epilepsy surgery evaluation, including scalp video EEG and intracranial EEG monitoring with stereotactic S-EEG intracranial monitoring, which resulted in greater delineation of the epileptogenic zone in the left central and paracentral frontal channels behind the posterior and mesial margins of the surgical cavity, likely with earlier onset on the mesial surface of left side of the interhemispheric fissure given early involvement of the right foot (**Figure 1**). Approximately 25 months after his initial surgery, he underwent a second scheduled left sided frontal craniotomy for resection of epileptogenic foci. He was continued on antiseizure medications postoperatively with subsequent self-reported improvement in seizure frequency. Since undergoing his second surgery, there has been notable reduction in seizure frequency from twice per week to twice per year from focal aware type triggered by medication reductions or alcohol consumption.

The case report is used as example to show the complexity of management for meningioma patient underwent craniotomy surgery. A separate IRB-approved project will be performed to analyze retrospectively the success rate of such procedures in our Center.

When it comes to the medical treatment of primary brain tumors (PBTs) in general, there is no robust, randomized studies to support the choice of ASMs. Several factors should be taken into considerations including gender, age, cost, profession, cognition, common medication related side effects, neurological baseline related to tumor/surgery (in order to avoid additive drug adverse events), medications pharmacokinetics, drug-to-drug interactions, efficacy and comorbidities. Other considerations are interaction with chemotherapy treatment and radiation effect on the brain. Some type of tumors (like low grade tumors) are known to be resistant to treatment with ASMs due to several hypothesis like intrinsic severity of the underlying mechanism of epileptogenicity, altered expression of molecules which ASMs work on, or changing of the expression of transporters at the blood-brain barrier limiting drug penetration to the epileptogenic tissue (61). Newer ASMs (oxcarbazepine, topiramate, lamotrigine, levetiracetam, zonisamide, and lacosamide) have provided better tolerability and efficacy due to different aspects including non-enzyme inducing property, limited drug to drug interaction, pure renal excretion, and lesser side effects. Older generation ASMs like carbamazepine, phenytoin and phenobarbital are falling out of favor due to high protein-bound, medications interaction and hepatic P-450 induction. Adverse events to ASMs are reported to be higher in PBTs than in the general epilepsy population (24% vs 0.5-12%) (57). In PBTs ASMs adverse events directed to the brain function such as executive function, attention span, cognitive function are six fold higher than the adverse events related to the radiation of the brain (62). Overall, the best risk–benefit ratio of which ASM to use is based on the physician’s judgment. It is very important to mention that treatment should be started after a single seizure. Based on the American Academy of Neurology (AAN) guideline, there is no need for prophylactic treatment with ASMs in patients with brain tumor, without history of seizures. It is also suggesting that tapering and discontinuing ASMs after the first postoperative week is appropriate if there is no history of seizures (57). In summary, the strategy of drug selection for the management of BTRE should favor drugs with parenteral administration, the ASMs which don’t need slow titration, and should avoid enzyme inducing ASMs. If monotherapy fails, consider combination therapy, poor compliance, repeated surgery and tumor recurrence/progression.

There is a wide range of reported efficacy of each individual ASM: oxcarbazepine as a monotherapy: 62.9%; topiramate as a monotherapy: 55.6%; Gabapentin, pregabalin, tiagabine, zonisamide as an adjunctive therapy: 27.4-100%, levetiracetam both in monotherapy and as add-on: 47.4% to 88%; lacosamide as an add-on drug with 42.9% (63). Levetiracetam and valproic acid are the most widely studied medications in tumor related epilepsy. Levetiracetam was studied against Valproic acid and the failure to treat seizures in glioblastoma was 33% vs 50% perhaps due to its tolerability and property of enhancing p53-mediated inhibition of methylguanine-DNA methyltransferase in this patient population (64). The most attractive factors for levetiracetam popularity are its well tolerability, its ease to use without a need for titration, no interaction with other ASMs, not hepatically metabolized by CYP450, and thus the absence of interaction with some chemotherapy drugs used in certain cases of BTRE, and finally good insurance coverage.

In a recent published survey of ASMs prescription preference among the European neuro-oncologists, levetiracetam is considered the first choice for brain tumor patients with the presumed highest efficacy and least adverse effects (65). ASMs are different in the pharmacokinetics, treatment efficacy, and side effects, which were reviewed by Maschio in detail (63).

Management of seizures should extend beyond pharmacological options. Untreated seizures can put patients at a risk of catastrophic outcome such as sudden unexpected death in epilepsy patients. Furthermore, seizures can negatively affect patient lifestyle including work, employment, education and driving. The risk of physical injury or death is not restricted to the driver and passengers, but applies to pedestrians and people in other vehicles. Different American States have different laws to determine which group of patients with epilepsy can drive. Seizures can result in other physical injuries. Patients with intractable epilepsy should be treated in tertiary centers where they can receive medical, social, and behavioral support and more importantly evaluation for epilepsy surgery.

## Future Insights

Despite advancements in understanding the pathophysiologic mechanisms and management of meningioma related epilepsy, important knowledge gaps remain. Pertinent questions include, “who are patients most at risk for seizures?” and “when to start ASMs and for how long?". The risk of persistent postoperative seizures underscores the need for further research on seizure control in meningioma patients. Long-term and arbitrary use of ASMs in meningioma patients emphasize the importance of guidelines for appropriate patient selection. Thus, prospective randomized trials are needed to guide ASMs selection and prescription. STOP ‘EM is an ongoing randomized controlled trial, with an end date of Sept 2027 (66). It aims at determining the need for ASMs postoperatively in seizure naïve patients. The study’s main goals are determining the efficacy of levetiracetam in seizure prevention over 12 months after surgery, the effect of starting levetiracetam on the ability to resume driving, quality of life, and cost-effectiveness.

## Conclusion

Understanding and predicting seizures in meningioma can help guide seizures control and allow for better determination of patients at risk before and after surgery. The current medical literature provides limited data for postoperative seizure prediction and optimal management in patients with meningioma related epilepsy. In reference to the cohort of meningioma patients undergoing surgery stratified based on preoperative seizure status to postoperative seizure status, it is logical to identify four different groups: no seizures to no seizures, seizures to no seizures, no seizures to seizures, and seizures to seizures. The future effort on stratifying patients into these four groups including medications alone, surgery/ies alone, medications + surgery/ies will be able to predict surgical outcome and optimally treat patients with the most successful modalities.

## Author Contributions

RE, HT, LH, WB and FB contributed to conception and design of the review. RE wrote the first draft of the manuscript. AA wrote sections of the manuscript. HT, LH, AA, WB and FB contributed to manuscript revision. All authors approved the submitted version.

## Conflict of Interest

The authors declare that the research was conducted in the absence of any commercial or financial relationships that could be construed as a potential conflict of interest.

## Publisher’s Note

All claims expressed in this article are solely those of the authors and do not necessarily represent those of their affiliated organizations, or those of the publisher, the editors and the reviewers. Any product that may be evaluated in this article, or claim that may be made by its manufacturer, is not guaranteed or endorsed by the publisher.
